# Nutritional Management of Irritable Bowel Syndrome

**DOI:** 10.3390/nu18040699

**Published:** 2026-02-22

**Authors:** Luigi Colecchia, Giovanni Marasco, David Meacci, Cesare Cremon, Alessandra Pivetti, Giulia Manni, Arianna Gobbato, Mira Xhuveli, Anna Rita Di Biase, Antonio Colecchia, Giovanni Barbara

**Affiliations:** 1 Department of Medical and Surgical Sciences (DIMEC), University of Bologna, 40126 Bologna, Italy; luigi.colecchia@studio.unibo.it (L.C.); giovanni.marasco4@unibo.it (G.M.); david.meacci@studio.unibo.it (D.M.); cesare.cremon@aosp.bo.it (C.C.); arianna.gobbato@studio.unibo.it (A.G.); mira.xhuveli@studio.unibo.it (M.X.); giovanni.barbara@unibo.it (G.B.); 2 IRCCS Azienda Ospedaliero-Universitaria Policlinico di Sant’Orsola di Bologna, 40126 Bologna, Italy; 3 Gastroenterology Unit, Azienda Ospedaliero-Universitaria di Modena, 41124 Modena, Italy; pivetti.alessandra@aou.mo.it (A.P.); giuliamannimed@gmail.com (G.M.); 4 Pediatric Department, Azienda Ospedaliero-Universitaria di Modena, 41124 Modena, Italy; dibiase.annarita@aou.mo.it; 5 CHIMOMO Department, University of Modena & Reggio Emilia, Via del Pozzo 71, 41121 Modena, Italy

**Keywords:** irritable bowel syndrome, low-FODMAP diet, gluten-free diet, starch- and sucrose-reduced diet, Mediterranean diet

## Abstract

Irritable bowel syndrome (IBS) is a common gastrointestinal disorder characterized by recurrent abdominal pain and altered bowel habits that significantly impair patients’ quality of life. Dietary triggers of IBS symptoms are common, and consequently, diet-based treatments are often prescribed. We conducted a review of current evidence on dietary interventions for IBS, focusing specifically on the evaluation of the scientific rationale and effectiveness of the most commonly adopted diets. Clinical trials and guideline recommendations were analyzed to assess each diet’s efficacy in symptom relief and patient adherence. Traditional dietary advice, although not a structured diet, but rather a set of lifestyle and dietary recommendations, is commonly recommended as first-line therapy and provides a solid base for symptom improvement in almost half of patients with IBS. Conversely, the low-FODMAP diet is a strict dietary pattern characterized first by the exclusion and then by the gradual and personalized reintroduction of several foods. Several clinical trials have demonstrated the efficacy of a low-FODMAP diet in reducing global IBS symptoms, and due to the established evidence, it is now incorporated into many clinical guidelines as a second- or even first-line approach for patients with IBS. Limited data supports the starch- and sucrose-reduced diet as an option for symptom relief, with evidence stemming from the relatively recent finding of hypomorphic variants of the sucrose-isomaltase gene in a subset of patients with IBS. Nonetheless, its application in clinical practice is still very limited. Data on gluten-free diet is more controversial as although it may benefit a subset of patients with IBS, strong evidence is still lacking for identifying the best candidates for a restrictive diet with a high burden in terms of economical, psychological and social costs. Beyond exclusion diets, a few studies on the Mediterranean diet suggest it may be a potential option with benefits that go beyond IBS symptom relief. Overall, dietary modification can significantly alleviate IBS symptoms. Tailoring recommendations to individual patient triggers may further enhance outcomes.

## 1. Introduction

Irritable bowel syndrome (IBS) is one of the most common disorders of gut–brain interaction (DGBI) and is described as recurrent abdominal pain associated with altered bowel habits [1,2]. The pathophysiology of IBS is multifactorial and not fully known. Different studies propose a variety of mechanisms, including visceral hypersensitivity, altered intestinal motility, gut–brain axis dysregulation, low-grade mucosal immune activation, genetic polymorphisms, psychosocial factors, food intolerances, and immune-mediated hypersensitivities [3,4,5,6]. Moreover, increasing evidence suggests that altered microbial taxonomy, shifts in microbial metabolite production and neuroactive compound synthesis may contribute to abnormal gut–brain signaling. These mechanisms have been proposed as potential biological links between IBS and extraintestinal comorbidities. In fact, individuals with IBS routinely face a significant reduction in quality of life due to health reasons, reporting chronic discomfort, psychological distress, and disruption of social functioning and work productivity, in addition to increased physician visits, diagnostic testing, and pharmacotherapy use [2]. Increased illness burden requires diagnostic and therapeutic approaches with the potential to improve symptoms and quality of life in patients with IBS.

Dietary management can be considered one of the first therapeutic approaches in patients with IBS, reflecting the common patient experience that the ingestion of food triggers or worsens symptoms [7,8]. Conventionally, clinicians have recommended general lifestyle modifications and traditional dietary advice (TDA) as first-line nutritional management for IBS [9,10,11]. These recommendations, based on consensus guidelines and clinical experience, include eating regular meals without skipping, avoiding large or fatty meals, limiting caffeine and alcohol, identifying and avoiding individual trigger foods, ensuring adequate hydration, and adjusting fiber intake (favoring soluble fiber while avoiding excess insoluble fiber). Over the past decade, other dietary management strategies have been tried. The low-FODMAP diet (LFD), characterized by the restriction of fermentable oligo-, di-, mono-saccharides and polyols (short-chain carbohydrates that are poorly absorbed and fermented in the gut), has been validated in multiple randomized controlled trials (RCTs), revealing symptomatic benefit in a significant proportion of patients with IBS, and it is now recognized as an efficacious therapy and adopted into several national clinical guidelines [9,10,11,12,13]. Another dietary approach that has recently gained attention is the gluten-free diet (GFD), as even in the absence of celiac disease (CD), wheat and gluten have been implicated in IBS symptom generation, potentially through overlapping non-celiac gluten or wheat sensitivity. However, overall evidence for GFD in IBS is controversial and investigations continue [14,15].

An emerging strategy is the starch- and sucrose-reduced diet (SSRD). This diet was originally developed in the context of congenital sucrase-isomaltase deficiency, but recent evidence suggests that even a subset of patients with IBS may have suboptimal sucrase-isomaltase activity contributing to symptoms [16,17,18]. The SSRD focuses on limiting complex starches and sucrose in the diet, thereby reducing the fermentable substrate load in the colon. Beyond exclusion diets, there is interest in broader healthy eating patterns such as the Mediterranean diet (MD) in IBS [19,20,21]. The MD, rich in plant-based foods, fiber, and unsaturated fats, is renowned for its benefits in general health and inflammation. Its application in IBS is a newer area of study, motivated by the idea that an anti-inflammatory dietary pattern could ameliorate symptoms without the need for stringent food eliminations. Data on comparison between diets’ effectiveness is still scarce and mostly relies on trials comparing new dietary advice with TDA; however, a recently published systematic review and network meta-analysis summarized the available data evaluating 28 RCTs and a total of 2338 patients testing 11 different dietary interventions for IBS. The strongest available evidence was found for the LFD, which consistently showed benefits for global IBS symptoms, abdominal pain, and bloating; GFD; MD; and SSRD, although with fewer studies [22]. Based on this recent data, we aimed to provide a detailed analysis of the most common dietary patterns used in IBS, along with the latest available evidence and current recommendations. Conceived as a narrative review of the literature, this paper conducted a structured search of major electronic databases (MEDLINE via Pubmed, Scopus and Embase) using predefined keywords related to irritable bowel syndrome and dietary interventions, with priority given to randomized controlled trials, meta-analyses, and current international guideline recommendations. Additional relevant cohort and mechanistic studies were included to support contextual interpretation of emerging evidence.

## 2. Traditional Dietary Advice

Traditional dietary advice is recommended as the first-line diet for IBS management in European [10], British [9], and Italian [11] guidelines (Table 1). Contrary to popular belief, TDA is not a structured diet but rather a series of dietary and lifestyle recommendations proposed in the National Institute for Health and Care Excellence (NICE) guidelines and by the British Dietetic Association (BDA) in the United Kingdom [23]. These recommendations emphasize balanced nutrition, regular eating habits, physical activity, and the exclusion or limitation of certain foods that may exacerbate symptoms. TDA includes eating at regular intervals, not skipping meals, chewing food slowly, and avoiding long periods between meals. Patients with diarrhea are instructed to avoid artificial sweeteners, while patients with constipation should maintain adequate hydration [23]. TDA also emphasizes limiting the intake of foods that may worsen IBS symptoms. For example, alcohol should be reduced to a minimum due to its association with increased intestinal permeability, altered gut microbiota, and impaired gastrointestinal motility [24,25]. Beverages containing caffeine such as coffee, tea, and energy drinks should be limited, as caffeine stimulates gastric acid secretion and enhances colonic motility, particularly in the distal region [26,27]. In addition, spicy and fatty foods are restricted due to their capacity to stimulate transient receptor potential vanilloid (TRPV1) channels—which are usually overexpressed in individuals with visceral hypersensitivity—and to modulate gastrocolic and small intestinal motility [28,29]. Patients with suspected milk sensitivity should limit dairy intake, while gas-producing foods (e.g., beans, cabbage, onions) should be avoided. Moreover, TDA promotes the intake of soluble fibers (e.g., linseeds, oats) while reducing insoluble ones, which are typically associated with the worsening of bloating and abdominal pain [23].

However, despite its diffuse application, the recommendation of TDA in IBS management is based more on clinical experience than robust evidence. Indeed, most dietary studies on IBS have used TDA as a control intervention, and few have directly assessed its efficacy. Moreover, most of the studies involving this dietary and lifestyle recommendations showed that TDA is as effective as the other diets in ameliorating IBS symptoms. As a matter of fact, an RCT comparing LFD to a modified TDA in 92 IBS-D patients over a 4-week period showed that the proportion of participants reporting adequate relief for ≥50% of the last two weeks of the period study was 52% in the LFD group and 41% in the TDA group (*p* = 0.31) [30]. Specifically, the LFD group showed greater improvements in most individual abdominal symptoms, such as abdominal pain, abdominal distension, stool consistency and urgency, while the TDA group achieved significant benefits in stool consistency and urgency. Other trials reported similar results; for example, Böhn et al., in a multicenter, parallel, single-blind study of 67 patients with IBS, observed that both LFD and TDA led to comparable reductions in the IBS-SSS score (≥50-point decrease in 50% of LFD vs. 46% of TDA patients, *p* = 0.72) after 4 weeks [31]. In contrast, other RCTs found the LFD to be more effective than TDA in patients with IBS-D. A more recent RCT compared the efficacy of TDA and LFD in 110 IBS-D patients. Both groups, after a 6-week period, showed improvement in IBS symptoms, but the LFD group exhibited a significantly greater reduction in specific IBS-SSS items, including abdominal pain intensity (*p* = 0.001), pain frequency (*p* = 0.0017), and abdominal distension (*p* < 0.001) [32]. These heterogeneous findings might be correlated to variability in how TDA was delivered across studies. Indeed, in some trials, patients following TDA were allowed to consume gas-producing foods (e.g., onions, cabbage, and legumes), while in others, these foods were restricted [33]. Despite this heterogeneity, TDA remains a well-tolerated, cheap and often accepted intervention, with a low risk of developing nutritional deficiencies or eating disorders. These characteristics make it suitable as a first-line dietary option for patients with IBS, especially when guided by experienced healthcare professionals.

**Table 1:** Dietary recommendations according to international guidelines.

Diet Guidelines	Italian [11]	United Kingdom [9]	European [10]	American [34]	Canadian [35]
Traditional dietary advice	First-line diet (recommendation: strong, quality of evidence: very low)	First-line diet *(recommendation: strong, quality of evidence: weak)*	First-line diet *(recommendation: not stated, quality of evidence not stated)*	Not explicitly recommended	Not mentioned
Low-FODMAP diet	Second-line diet *(recommendation: conditional, quality of evidence: low)*	Second-line diet *(recommendation: weak, quality of evidence very low)*	Second-line diet *(recommendation: strong, quality of evidence low)*	First-line diet *(recommendation: conditional, quality of evidence: very low)*	First-line diet *(recommendation: conditional, quality of evidence: very low)*
Gluten-free diet	Recommendation against its use *(recommendation: strong, quality of evidence: very low)*	Recommendation against its use *(recommendation: weak, quality of evidence very low)*	Recommendation against its use *(recommendation: strong, quality of evidence low)*	Not mentioned	Recommendation against its use *(recommendation: conditional, quality of evidence: very low)*
Mediterranean diet	Not mentioned	Not mentioned	Not mentioned	Not mentioned	Not mentioned
Starch and sucrose-reduced diet	Not mentioned	Not mentioned	Not mentioned	Not mentioned	Not mentioned

## 3. Low-FODMAP Diet

FODMAP is an acronym for Fermentable Oligosaccharides, Disaccharides, Monosaccharides and Polyols, a group of short-chain carbohydrates that are poorly absorbed and not entirely digested in the human small intestine [36]. This group includes fructans, fructo-oligosaccharides (FOS), galacto-oligosaccharides (GOS), polyols (such as sorbitol and mannitol), and lactose [37]. Due to the lack of appropriate digestive enzymes in humans, FODMAPs are fermented by colonic microbiota, leading to the production of gases such as hydrogen, methane, and carbon dioxide [38]. This fermentation can result in luminal distension and symptoms like abdominal pain, bloating, and flatulence [38]. Furthermore, FODMAPs are osmotically active compounds capable of attracting water into the intestinal lumen, which can increase stool liquidity, potentially leading to diarrhea and additional distension-related discomfort [39]. While these effects occur in all individuals, they do not always lead to symptoms. However, in susceptible individuals, such as those with increased intestinal permeability or visceral hypersensitivity—as is often the case in patients with IBS—these effects may be exaggerated, contributing to significant symptom burden and impaired quality of life [40,41,42]. In 2005, Monash University proposed the LFD as a potential dietary intervention to alleviate gastrointestinal symptoms in patients with IBS, aiming to reduce the intake of fermentable carbohydrates that may trigger symptoms. Due to the complexity of the diet and potential nutritional risks, it should be implemented under the supervision of a qualified dietitian [43]. Two main strategies are used to implement the LFD: the top-down and the bottom-up approach [44].

The top-down approach is the most commonly employed and consists of three distinct phases. The first one is the “restriction phase”, which usually has a duration of 4–6 weeks. All high-FODMAP foods are excluded from the diet. This includes common triggers such as garlic, onion, mushrooms, apples, peaches, and pears. Most dairy products are restricted, with notable exceptions like lactose-free milk. Protein sources should mainly comprise plain-cooked meats and eggs, while legumes and processed meats are avoided. Cereal-based products containing wheat, rye, or barley are replaced with gluten-free, rice-based, or oat-based alternatives [36,45]. Then, a “reintroduction phase”, usually of 6–8 weeks, can be approached. Previously excluded foods are systematically reintroduced one at a time, typically over three-day intervals, to identify specific FODMAP subtypes that elicit symptoms [44,46]. Foods that trigger adverse symptoms are identified and excluded from the long-term diet. The last phase is the “personalization phase”, which is a long-term maintenance phase that includes only tolerated high-FODMAP foods and all low-FODMAP ones. The goal is to ensure nutritional adequacy while maintaining symptom control [36,46]. 

Alternatively, the bottom-up approach (also known as the “gentle FODMAP” strategy) involves initially restricting only a subset of commonly problematic high-FODMAP foods—such as onion, garlic, apples, legumes, milk, and wheat—with additional exclusions made only if symptoms persist [44,47]. Although this approach also follows a three-phase structure (restriction, reintroduction, personalization), it is less restrictive than the top-down method and often better tolerated by patients. However, overall, limited data are available regarding the clinical effectiveness of the “gentle FODMAP” approach in IBS populations [44,47]. 

The efficacy of LFD in patients with IBS is well documented in the literature. Multiple studies have demonstrated its effectiveness in alleviating IBS-related symptoms. Notably, the CARIBS study [12] in 2024 evaluated the effects of an LFD combined with traditional dietary advice (TDA), comparing it to a low-carbohydrate diet and optimized pharmacological therapy. This study enrolled 294 subjects: 96 were assigned to an LFD-TDA combined group, 97 to a low-carbohydrate diet, and 101 to optimized medical treatment. After four weeks of treatment, all three groups showed a significant reduction in the IBS Symptom Severity Scale (IBS-SSS) score. However, the combined LFD + TDA approach proved to be superior to both the low-carbohydrate diet and optimized medical therapy in symptom improvement. In particular, a higher percentage of patients in the LFD-TDA group achieved a reduction of at least 50 points in IBS-SSS score, compared to baseline (76% vs. 71% vs. 58%, respectively, for LFD-TDA, low-carbohydrate diet, and optimized medical therapy groups, *p*-value = 0.023) [12]. In addition to gastrointestinal benefits, the dietary intervention was also associated with improvements in psychological well-being, including reduced levels of anxiety and depression among study participants [12,48]. Further supporting evidence comes from a recent pilot randomized controlled trial, which compared the “gentle” LFD with the conventional LFD in patients with IBS. Despite the limited sample size (n = 32), the study demonstrated that the “gentle” LFD was equally effective in reducing abdominal pain, suggesting potential for broader clinical application due to its improved tolerability and flexibility [49]. Moreover, LFD resulted particularly effective in patients with specific alterations in microbiota composition, potentially leading to personalized therapy in patients with IBS. Vervier et al. performed a metagenomic analysis of stool microbiota in 56 IBS non-constipated patients and a comparable number of healthy controls leading to the definition of two different microbiological profiles in patients with IBS [50]. IBS-P (pathogenic-like) microbiomes were associated with a higher abundance of *Firmicutes*, a reduction in *Bacteroides* species, and higher expression of genes involved in SCFAs synthesis. Conversely, IBS-H (healthy-like) microbiota composition was similar to that of healthy subjects. Both IBS-P and IBS-H patients followed a 4-week period of LFD. At the end of the interventions, the IBS-SSS score was significantly reduced in both groups, compared to baseline. However, the degree of response was higher in IBS-P patients (ΔIBS-SSS IBS-H = 114 vs. in IBSP = 194, *p* = 0.02). After the 4-week period diet, another microbiota analysis showed that microbiota profiles in IBS-P patients became more similar to those of IBS-H, with an increased abundance of *Bacteroides* and a reduction in several pathobiont levels [50]. Possible mechanisms of action behind LFD’s effect have been explored in an RCT in which patients with IBS were randomized to either a low- (n = 20) or high-FODMAP diet (n = 20) for 3 weeks to assess metabolome and microbiome changes [51]. Interestingly, the authors found that LFD increased *Actinobacteria* richness and diversity, decreasing the relative abundance of bacteria involved in gas consumption, likely contributing to symptoms. Moreover, histamine urinary levels were significantly reduced after LFD, likely due to reduced inflammation at the level of the gut [51]. Further indirect evidence of LFD’s effect in modulating gut immunity and inflammation can be gathered from a single-arm study on 20 IBS-D patients in which both proinflammatory cytokines and gut permeability markers were assessed before and after a 12 weeks course of LFD [52]. In fact, an improvement in intestinal mucosal integrity (assessed through the urinary recovery of non-absorbable sugars, serum and fecal zonulin levels, intestinal fatty acid-binding protein, and diamine oxidase serum concentrations) was observed, along with a significant reduction in proinflammatory cytokines and lipopolysaccharide levels.

The LFD is currently recommended as first-line dietary therapy for IBS in American [34] and Canadian [35] guidelines. In contrast, European [10], British [9], and Italian [11] guidelines recommend it as a second-line option, typically following a failure of TDA. Recent studies have also compared the efficacy of LFD in reducing IBS symptoms against a commonly prescribed antispasmodic drug—otiloinium bromide (OB)—in 459 patients with IBS followed at a primary care level in the United Kingdom [53]. The authors found that the response rate after 8 weeks was significantly higher with diet compared with OB (71% (155/218) vs. 61% (133/217), *p* = 0.03). A recent umbrella review of 16 different meta-analyses, including more than 9000 patients, showed that the LFD is useful in reducing IBS symptoms, as evidenced by a reduction in IBS-SSS scores (standardized mean difference (SMD) = −0.599) and by improvements in quality of life. However, no significant effects on abdominal pain, stool consistency, or stool frequency were reported, suggesting that the absolute effect on specific symptoms may be modest or limited in certain patients [54]. Moreover, most studies assessing the effectiveness of the LFD in IBS are unblinded due to the strict dietary intervention required by this diet. This may lead to performance bias, as awareness of the treatment can result in greater attention to the LFD group and different behaviors among enrolled participants [54,55]. Nonetheless, LFD is not recommended as a first-line approach in several countries due to several limitations in the current body of evidence. Notably, most studies focus only on the elimination phase, with limited data available on the long-term safety and efficacy of the full three-phase LFD protocol [56]. As an additional limitation, studies on the LFD show a high degree of heterogeneity not only in how the diet is administered (type of approach, duration of the different regimens, and correct sequencing of the three phases) but also in the characteristics of the investigated populations, the choice of comparison dietary regimens, and the outcomes measured [57]. Other concerns include the cost, difficulty in long-term adherence, and accessibility of the diet, which may not be feasible for all patients [14], as well as potential negative effects on the gut microbiota, particularly a reduction in beneficial bacterial populations such as *Bifidobacteria* [58]. Furthermore, long-term adherence to the LFD has been associated with decreased energy intake and nutrient deficiencies in some individuals [59]. For instance, Staudacher et al. compared the efficacy of LFD with that of a habitual diet in patients with IBS. Participants adhering to the restricted dietary regimen showed a reduction in GI symptom severity along with a reduction in *Bifidobacteria* abundance and SCFA fecal levels and a significative lower intake of carbohydrates, starch, calcium, and iron [60]. Other studies have shown that the LFD has been associated with vitamin deficiencies (such as vitamin C), reduced antioxidant levels, polyphenol depletion, and excessive weight loss [59]. Conversely, Bellini et al. showed that an 8-week LFD in 26 patients with IBS, supervised by a skilled nutritionist, alleviates IBS symptoms without compromising nutritional status as assessed with a blood test and bioelectrical impedance analysis [61]. These findings suggest that strict monitoring by an expert nutritionist might be crucial in achieving IBS symptoms improvement without exposing patients to the risk of nutritional deficiencies. Another emerging concern is the potential risk of disordered eating behaviors among patients with IBS following the LFD. Given the higher prevalence of psychological comorbidities in this population, strict dietary patterns may contribute to the development of conditions such as avoidant/restrictive food intake disorder (ARFID) and orthorexia nervosa [62,63].

## 4. Gluten-Free Diet

Up to 20% of the general non-celiac population, mostly encompassing patients with IBS, report that eating wheat or gluten triggers gastrointestinal symptoms and, accordingly, they often self-prescribe GFD [64,65]. The involvement of wheat in the pathophysiology of IBS remains complex and incompletely understood, with substantial overlap between IBS and non-celiac gluten/wheat sensitivity [66]. Components of wheat, including gluten, FODMAPs such as fructans and FOS and non-gluten proteins such as *α*-amylase-trypsin inhibitors (ATIs), are often thought to drive symptom generation in IBS, but data are highly controversial [67,68,69]. In a recently published double-blind RCT, 29 patients with IBS first completed a gluten-free diet run-in phase and were subsequently challenged, under double-blind conditions, with cereal bars containing wheat, isolated gluten, or sham, administered in a randomized sequence. In the 28 patients who completed the study, the authors found no statistically significant differences in the proportion of participants with a worsening of IBS-SSS of at least 50 points after receiving wheat or gluten versus sham [15]. The relationship between gluten and other wheat components is further complicated by the presence of fructans, a type of FODMAPs, which can increase luminal water content and gas production, exacerbating symptoms in susceptible patients with IBS [70].

Regarding GFD efficacy in IBS, a crossover RCT on 20 patients with IBS and 21 healthy controls demonstrated that patients with IBS had significant reductions in IBS-SSS scores and fewer loose stools during GFD compared to gluten-containing diets and that responders displayed distinct gut microbial and metabolomic signatures [71]. These data, has been confirmed by a landmark RCT comparing GFD, LFD, and TDA in non-constipated patients with IBS, which found high clinical response rates across all groups: 58% for GFD, 55% for LFD, and 42% for TDA. However, the significantly higher burden in terms of cost, social limitations, and psychological stress of GFD compared to TDA limits its applicability [14,68,69]. Overall, these findings suggest that GFD may improve symptoms in a subset of patients with IBS, particularly those with diarrhea-predominant phenotypes; however, the effect is likely mediated by the reduction in other components present in gluten-containing foods, mostly fructans and other FODMAPs, rather than gluten itself.

Lastly, long-term risks of GFD are present. In fact, experiences with CD have revealed that an inadequately balanced GFD can lead to increased relative intake of lipids and simple carbohydrates with respect to proteins and complex carbohydrates, increasing the risk of obesity, diabetes, and metabolic syndrome [72,73]. Moreover, the GFD carries a long-term risk of micronutrient imbalances. Specifically, the daily intake of vitamin D, vitamin B12, iron, zinc, magnesium, calcium, and dietary fibers can be insufficient, leading to complications such as increased rates of osteopenia, osteoporosis, anemia, or constipation [73].

## 5. Mediterranean Diet

The Mediterranean diet (MD) represents a traditional dietary pattern prevalent in countries bordering the Mediterranean Sea. It is characterized by high consumption of whole grains, fruits, vegetables, nuts, and seeds. Fish and other seafood, poultry, and dairy are eaten in moderation. Red meat and foods high in sugar are eaten on occasion. Olive oil is the principal source of dietary fat [74].

The MD has been associated with numerous health benefits, particularly in cardiovascular and metabolic disorders, as well as cognitive disorders and overall mortality [75]. Moreover, several studies have demonstrated that MD can prevent and ameliorate specific psychological disorders, including major depressive disorder [76,77]. Multiple mechanisms have been postulated to account for these effects, including the production of microbial-derived anti-inflammatory molecules and neuroactive compounds [78]. Furthermore, it has been hypothesized that the MD may exert beneficial effects on gastrointestinal symptoms through the modulation of the gut microbiota, reducing gut mucosal inflammation, reinforcing gut barrier function and regulation of the gastrointestinal motility [79,80,81]. In fact, adherence to MD has been associated with an increased abundance of *Firmicutes* and *Bacteroidetes* taxa along with a significative increase in SCFA production. These molecules, known for their anti-inflammatory properties, may contribute to the amelioration of neuroinflammation, which is characteristic of several psychological and neurological disorders [77,82]. Due to these potential health benefits, MD has been investigated as a possible dietary regimen in patients with IBS [83].

Although the MD has been proposed as a potential therapeutic diet for IBS treatment, its clinical efficacy is still unclear [21]. Initial data emphasizing the role of MD in IBS treatment were derived from a recent RCT by Staudacher et al. [20]. In this study, 59 patients with IBS were assigned to follow either the MD or their habitual diet for a 6-week period. At the end of the study, the MD group showed a significantly lower IBS-SSS score compared with the control group (168 vs. 260; *p* < 0.001), as well as a greater reduction from baseline (−113 vs. −20; *p* < 0.001). Notably, the MD group also demonstrated a significant decrease in anxiety and depression, evaluated using the Hospital Anxiety and Depression Scale (HADS), and in specific domains of the Gastrointestinal Symptom Rating Scale (GSRS), including abdominal pain and bloating. Conversely, microbiota analysis did not show significant differences in gut microbial composition between the two groups. This finding might suggest that longer intervention periods are necessary to induce measurable changes in microbiota composition [20]. However, in a recently published well-conducted RCT by Chen et al., assessing the role of MD in 106 patients with IBS and 108 controls, the authors did not find a correlation between MD adherence and IBS symptoms. Conversely, they found that the consumption of certain MD foods was associated with greater severity of IBS symptoms, as confirmed via multivariate analysis. Adherence to a symptom-modified MD was, however, associated with favorable gut microbiota changes [84]. These findings suggest that MD, likely due to the presence of FODMAPs within the diet, may not be suitable for all patients with IBS; however, modified MD based on patients’ symptoms may be a valuable option. Very recent studies compared, in a randomized fashion, MD and TDA in 139 persons with IBS from across the United Kingdom [85]. Clinical response, defined as a 50-point or greater reduction in IBS-SSS was achieved in 62% (95% confidence interval (CI): 50–73%) following MD versus 42% (95% CI: 31–55%) following TDA. Similarly, another recent RCT compared the MD with the LFD in non-constipated patients with IBS over a 4-week period. Both diets were effective in reducing abdominal pain. However, LFD was more impactful in ameliorating other IBS-related symptoms, such as bloating and abnormal stool consistency [21]. Finally, a 2019 prospective cross-over trial from Paduano et al. compared the efficacy of three different diets in ameliorating IBS symptoms. In total, 34 subjects were assigned to follow the LFD, then the GFD, and lastly the MD, each for a 4 week-period. All three dietary regimens significantly reduced abdominal pain (*p* < 0.01) and bloating (*p* < 0.01) compared to baseline levels, while improving quality of life (*p* < 0.05). However, the MD was the most tolerated and favored dietary approach, increasing the likelihood of long-term adherence [19].

## 6. Starch- and Sucrose-Reduced Diet

Congenital sucrase-isomaltase deficiency (CSID) is an autosomal recessive disorder characterized by the lack of functional sucrase–isomaltase (SI), a brush border enzyme required for digesting sucrose and part of ingested starch [86]. Affected infants often develop severe osmotic diarrhea, bloating, and failure to thrive when introduced to sucrose- or starch-containing foods [86]. In Western populations, CSID is quite rare, affecting around 0.2% of Europeans [86]. This condition is typically undiagnosed as the diagnostic gold standard is direct SI enzyme assay on small-bowel biopsy, but noninvasive tests (sucrose tolerance or hydrogen breath tests) and SI gene sequencing are available. Recent genetic studies have identified *hypomorphic* (partial loss-of-function) SI gene variants in adult patients with IBS, especially those with diarrhea-predominant IBS [87,88]. In a landmark study, known CSID mutations (in heterozygous state) were significantly more common in IBS cohorts compared to general population (respectively, 2.1% vs. 1.3%; OR: 1.57, *p* = 0.02) [17]. Subsequent studies showed that heterozygous mutations reduced SI enzymatic activity by 35%, causing reduced digestion of sucrose and starch, leading to higher stool frequency and altered gut microbiota [88]. Collectively, these findings suggest that even partial SI dysfunction can contribute to IBS symptoms, indicating that SI hypofunction is a novel pathogenic mechanism in a subset of patients [10,11]. Given SI’s role in digesting sucrose and starch, reducing these substrates could be considered a logical therapy in SI impairment [89,90]. SSRD eliminates most sugars and high-starch foods, while allowing non-SI digested carbohydrates. The diet is focused on decreasing the intake of sucrose contained in several fruits such as apples, apricots, bananas, dates, mangoes, oranges, peaches, and pineapples and starches contained in beets, legumes, carrots, onions, potatoes, and yams, but not sources of sugars such as in confectionaries, soda, and processed foods. Other fruits and vegetables such as avocadoes, berries, figs, kiwi, lemons, papaya, prunes, crucifers, eggplant, peppers, lettuce, spinaches, and zucchini are allowed along with fiber, fat, and protein in the form of all meats and fish, natural dairy products, eggs, nuts, and seeds [91]. By cutting sucrose and digestible starch, SSRD prevents the colonic accumulation of SI substrates that would undergo fermentation in SI-deficient patients, causing gas and osmotic diarrhea. This direct pathophysiological targeting contrasts with low-FODMAP diets, which restrict many short-chain carbs but do *not* specifically eliminate sucrose or regular starches.

Early pilot trials of SSRD in patients with IBS showed a high adherence and improved gastrointestinal symptom scores and quality of life. A prospective RCT on 105 patients with IBS, randomized in a 3:1 fashion to either SSRD or habitual diet, showed that after 4 weeks the intervention group displayed lower total IBS-SSS, “abdominal pain”, “bloating/flatulence”, and “intestinal symptom” influence on daily life scores compared to controls, and a 74%, responder rate, defined as a 50 point reduction in IBS-SSS scores, compared to 24% in controls [90]. The efficacy of SSRD was also tested against other routinely used dietary advice. In a non-inferiority 4-week RCT of SSRD versus LFD in 155 patients with IBS, randomized 1:1, 79% of SSRD patients achieved ≥50-point reduction in IBS-SSS at 2–4 weeks, not significantly different from a low-FODMAP approach [16]. All individual IBS symptoms (abdominal pain, bloating, diarrhea, urgency) with the exception of constipation, significantly improved on both SSRD and LFD. Notably, about 25% of both patients randomized to SSRD and LFD became essentially symptom-free (<75 IBS-SSS) after 4 weeks. Furthermore, extraintestinal symptoms (e.g., headaches, fatigue) also declined substantially on SSRD. The trial showed high patient adherence to SSRD, with 93.5% of patients completing the intervention after 4 weeks. Over a 6-month follow-up in which patients were free to return to ordinary diets or switch between LFD and SSRD, adherence naturally declined, but dropout rates tended to be lower with SSRD than with LFD [16]. A successive evaluation on the same study population showed that compared to LFD, SSRD patients lost more weight, decreased their BMI, and reported less sugar craving. In fact, SSRD influenced multiple hormonal and metabolic parameters [18]. A 4-week SSRD intervention caused significant reductions in insulin, C-peptide, gastric inhibitory peptide, and leptin, ghrelin, and adipokines, likely due to the reduced amount of dietary sugar intake. Lastly, SSRD induces distinctive microbial alterations [91]. In a randomized clinical trial conducted on a Swedish population, 4 weeks of SSRD significantly altered fecal microbiota composition: β-diversity changed (reflecting a different community structure) even though overall richness (α-diversity) was unchanged. Specifically, SSRD increased the relative abundance of *Proteobacteria* and decreased *Bacteroidetes*, and both changes were associated with lower IBS-SSS scores. At finer taxonomic levels, SSRD raised levels of certain Firmicutes genera (e.g., *Eubacterium eligens*, *Lachnospiraceae* UCG-001, *Lachnospira*) and lowered others (e.g., *Marvinbryantia*, *Olsenella*), known carbohydrate fermenters [92]. These data suggest that SSRD may be effective also by shifting microbial patterns by reducing the substrate and, consequentially, the relative abundance of bacteria that ferment starch and sucrose-forming gas. Not all patients, however, respond equally to SSRD. In combined Swedish and Spanish cohorts, all patients with IBSc carrying two hypomorphic SI alleles (“double-carriers”) experienced symptom relief on SSRD, whereas only ~74% of single-carriers or non-carriers did. In subgroup analysis, IBS-D patients showed the greatest improvement in diarrhea symptoms compared to IBS-C or IBS-M subgroups [89]. Overall, SSRD would be an ideal therapy in patients with known SI reduced function; however, due to the lack of routine genetic testing in IBS, the small sample size of published clinical trials, mostly from limited geographical areas, does not allow the recommendation of this diet until more generalizable data is available. Lastly, no data is available on the long-term sustainability and nutritional consequences of this nutritional regimen (Table 2).

## 7. Other Diets

Several additional dietary regimens have been investigated as potential therapeutic approaches for IBS. However, due to the limited data available regarding their effectiveness in ameliorating IBS symptoms, most of the current guidelines do not recommend or mention their routine implementation.

### 7.1. Low-Lactose Diet

Lactose is a disaccharide composed of glucose and galactose that is usually hydrolyzed by lactase, an enzyme expressed by enterocytes, and subsequently absorbed in the small intestine [92]. When lactose reaches the colon, it is fermented by the local microbiota, leading to gas production and abdominal pain. Furthermore, due to its osmotic properties, undigested lactose increases the amount of luminal water, potentially leading to diarrhea [93]. Although lactose intolerance is commonly reported among patients with IBS, true lactase deficiency (LD) is concretely determined only in a minority of cases [94]. A few studies have investigated the effect of a low-lactose diet (LLD) on IBS [95]. Krieger-Grübel et al. conducted a randomized crossover trial comparing the efficacy of LLD with that of LFD in 29 patients with IBS over an 11-week period [96]. Prior to the intervention, a lactose breath test was performed, which identified six patients with lactase deficiency; however, the enzyme deficit was only partial, and these patients did not exhibit significant differences in symptom patterns from those with normal test results. At the end of the study, both dietary regimens achieved a similar reduction in the IBS-SSS score compared with the baseline, without significant differences between the groups (mean IBS-SSS reduction of 63 and 51 points from the baseline, respectively, for LFD and LLDs, *p* = 0.85). Notably, a significant daily reduction in abdominal pain and bloating was observed only during the LFD period and not with the LLD regimen.

### 7.2. Fructose-Reduced Diet

Fructose malabsorption (FM) has been proposed as a possible mechanism of symptom genesis in patients with IBS [97]. This condition is caused by an incomplete absorption of fructose, a monosaccharide, across enterocytes in the small intestine, due to an impaired function of GLUT2 and GLUT5 transporters [98]. Thereby, the unabsorbed fructose that reaches colonic lumen undergoes fermentation by local microbiota, leading to abdominal pain, bloating, and altered bowel habits [99]. Fructose malabsorption, estimated to affect approximately one-third of patients with IBS, is diagnosed using fructose breath tests [100]. Therefore, some studies have investigated the possible role of fructose-reduced diet in reducing gastrointestinal symptoms in patients with IBS. In a pilot study by Sheperd et al., 26 patients with IBS and confirmed FM (via positive fructose breath test), who had previously responded to an LFD, were divide into four groups, each challenged for two weeks with beverages containing fructose, fructans, a combination of both, or glucose [101]. At the end of the intervention, adequate gastrointestinal symptom control was reported in only 30%, 23, and 21% in the fructose, fructans, and combination groups, respectively, against the 86% in the glucose control arm. Despite this result, a subsequent RCT in 2013 compared the efficacy of fructose-reduced diet with that of an LLD in a cohort of 320 patients with IBS over a 3-week period [102]. Both interventions demonstrated a significant reduction in abdominal symptom severity compared to the baseline (a mean decrease of 127 points based on the VAS score). Breath testing revealed that 111 patients had FM, 12 exhibited LM, and 72 exhibited both of these conditions. However, a post hoc analysis failed to demonstrate a benefit for selecting either dietary regimen for patients with specific enzyme deficiencies.

### 7.3. Tritordeum-Based Diet

Emerging evidence has suggested a possible role for tritordeum-based diet (TBD) as a possible dietary approach for IBS gastrointestinal and global symptom management. Tritordeum is a cereal derived from the hybridization of durum wheat with wild barley, which contains lower levels of fructans, gliadin, and carbohydrates compared to normal wheat. Its consumption has been associated with an increased abundance of beneficial fermenting bacteria and enhanced production of SCFAs and IL-10 [8,103]. Russo et al. compared the effects of a TDB and LFD in a randomized 12-week study involving 42 IBS-D patients randomized in a 1:1 fashion [104]. Both interventions were effective in improving IBS symptoms based on the IBS-SSS score reduction from the baseline (−130.5; 95% CI: 73.2 to 187.7 and−132.1; 95% CI: 74.9 to 189.4, respectively, for TBD and LFD). Neither diet altered micronutrient profiles; conversely, both significantly reduced fasting glucose and CRP values (*p* < 0.05) and increased vitamin D serum levels (*p* = 0.04). Another study involving women with IBS-D not only confirmed the potential effect of TBD in ameliorating abdominal symptoms, such as abdominal pain and bloating but also demonstrated a significant enhancement in psychological well-being [105]. A 12-week TBD led to a significative improvement in the IBS quality of life (IBS-QoL) total score compared with the baseline (from 68.54 ± 3.12 to 85.77 ± 1.97, *p* > 0.0001) and in multiple domains of the 36-Short Form Survey (SF-36), including mental health and vitality. Nonetheless, larger RCTs are warranted to further define the therapeutic potential of TBD in IBS management.

### 7.4. ARFID Risk and Implementation Diets

Avoidant/restrictive food intake disorder is defined by a persistent failure to meet nutritional and/or energy needs, resulting in significant weight loss, significant nutritional deficiencies, reliance on enteral feeding or oral nutritional supplements, or substantial interference with psychosocial functioning. Unlike other eating disorders, ARFID is not driven by concerns about body weight or shape [106]. Instead, it is typically caused by lack of interest in eating, sensory-based avoidance, and/or fear of aversive consequences such as choking, vomiting, or abdominal pain. Higher rates of this condition have been reported in achalasia, celiac, eosinophilic esophagitis, and inflammatory bowel disease [107]. A recent cross-sectional study on 4002 adults, of whom 1704 (42.6%) had symptoms compatible with at least one DGBI, found that the prevalence of ARFID-positive screens was significantly higher among participants with DGBI compared with those without DGBI (34.6% vs. 19.4%; OR: 1.67; 95% CI, 1.43–1.94) [108]. The presence of ARFID increased with the number of anatomical areas affected by a DGBI, ranging from 19.4% in those with no DGBI to 61.4% for DGBI in four anatomical areas (*p* < 0.001). The underlying mechanisms leading to food avoidance likely stems from the fear of symptom flare-ups. Effective dietary strategies help prevent ARFID in IBS patients; in fact, overly restrictive diets (e.g., long-term stringent low-FODMAP without professional dietary guidance) can exacerbate nutritional deficiencies and psychological distress, increasing the risk of ARFID-like behaviors [109]. To counter this, clinicians should emphasize a balanced, personalized diet plan that maintains nutritional variety while controlling triggers. Gradually reintroducing foods (as in the structured reintroduction phase of the low-FODMAP diet) can improve tolerance and confidence in eating. For example, by tailoring fiber intake and other dietary modifications to individual tolerance, healthcare providers help patients with IBS-C achieve symptom relief without unnecessary food avoidance. In fact, high-fiber diets are a first-line approach for chronic constipation, but fiber type is crucial [110]. Partially soluble fibers (e.g., psyllium husk or ispaghula) fermented by gut bacteria, dissolve in water to form a gel, softening stools. In contrast, insoluble fibers (e.g., cellulose and methylcellulose) are poorly fermented and can increase stool bulk. Although limited high-quality data is available, clinical trials support the use of soluble fiber rather than insoluble fibers for the management of constipation. Notably, however, side effects such as bloating and flatulence are common [111]. In addition, some fermentable fibers are also classed as prebiotics, such as galactooligosaccharides (GOS) or FOS [112]. Prebiotic fibers are known for being substrates for SCFA-producing bacteria such as *Bifidobacterium* and *Lactobacillus* and selectively stimulate their growth [113]. Nonetheless, their impact on IBS is controversial [112,113,114]. This is because carbohydrate fermentation can lead to gas production, potentially exacerbating symptoms in individuals with DGBIs. In fact, some prebiotics such as FOS contain fructose, which can cause bloating, flatulence, and abdominal pain [115,116]. In contrast, GOS has shown potential for symptom improvement in IBS, reaching the highest beneficial effects without exceeding 3.5 g/day, as larger amounts can induce gas and discomfort [8,117,118,119].

## 8. Conclusions

In conclusion, nutritional strategies have demonstrated potential benefits in symptom control and should be considered part of a multidimensional approach to patients with irritable bowel syndrome. Nonetheless, further data are needed to understand how different dietary approaches should be individualized to optimize symptom management in each patient.

## Figures and Tables

**Table 2:** Main examples of foods to avoid according to the most commonly prescribed diets for irritable bowel syndrome.

Traditional Dietary Advice	Low-FODMAP Diet	Gluten-Free Diet	Mediterranean Diet	Starch and Sucrose-Reduced Diet
Fried/fatty foods; spicy foods; alcohol; coffee; cola; carbonated drinks; lactose-rich dairy if intolerant; beans; lentils; cabbage; broccoli; onion; garlic; high-fructose fruits (e.g., apples; pear; watermelon); trigger foods; honey; sorbitol; xylitol; and ultra-processed foods.	Wheat; barley; rye; onion; garlic; leek; artichokes; asparagus; cauliflower; mushrooms; legumes; apples; pears; mango; cherries; prunes; watermelon; honey; sorbitol; mannitol; xylitol; and lactose-rich dairy.	Wheat; barley; rye; malt; gluten-containing sauces; and beer.	No strict exclusions. Excess fried/processed foods; too many sweet fruits; sugary desserts; sodas; packaged snacks; excess red meat; excess dairy and eggs.	Apples; apricots; bananas; dates; mangoes; oranges; peaches; pineapples; starches contained in beets; legumes; carrots; onions; potatoes; yams; and other sources of sugars such as confectionaries; soda; and processed foods.

## Data Availability

Not applicable.

## Abbreviations

The following abbreviations are used in this manuscript:

IBS	Irritable Bowel Syndrome
DGBI	Disorders of Gut–Brain Interaction
TDA	Traditional Dietary Advice
LFD	Low-FODMAP Diet
FODMAP	Fermentable Oligosaccharides, Disaccharides, Monosaccharides and Polyols
RCT	Randomized Controlled Trial
GFD	Gluten-Free Diet
CD	Celiac Disease
SSRD	Starch- and Sucrose-Reduced Diet
MD	Mediterranean Diet
IBS-SSS	Irritable Bowel Syndrome Symptom Severity Scale
NICE	National Institute for Health and Care Excellence
BDA	British Dietetic Association
TRPV1	Transient Receptor Potential Vanilloid 1
SCFAs	Short-Chain Fatty Acids
OB	Otilonium Bromide
ATIs	α-Amylase–Trypsin Inhibitors
CSID	Congenital Sucrase-Isomaltase Deficiency
SI	Sucrase-Isomaltase
OR	Odds Ratio
LLD	Low-Lactose Diet
FM	Fructose Malabsorption
GLUT	Glucose Transporter
TBD	Tritordeum-Based Diet
IBS-QoL	IBS Quality of Life Questionnaire
ARFID	Avoidant/Restrictive Food Intake Disorder
FOS	Fructooligosaccharides
GOS	Galactooligosaccharides
